# Accelerated dynamic magnetic resonance imaging from Spatial-Subspace Reconstructions (SPARS)

**DOI:** 10.1371/journal.pone.0317271

**Published:** 2025-01-31

**Authors:** Alexander J. Mertens, Hai-Ling Margaret Cheng

**Affiliations:** 1 The Edward S. Rogers Sr. Department of Electrical and Computer Engineering, University of Toronto, Toronto, Canada; 2 Ted Rogers Centre for Heart Research, Translational Biology & Engineering Program, Toronto, Canada; 3 Institute of Biomedical Engineering, University of Toronto, Toronto, Canada; Ulsan National Institute of Science and Technology, REPUBLIC OF KOREA

## Abstract

Dynamic contrast-enhanced (DCE) magnetic resonance imaging (MRI) ideally requires a high spatial and a high temporal resolution, but hardware limitations prevent acquisitions from achieving both simultaneously—either high temporal resolution is exchanged for spatial resolution, or vice versa. Even state-of-the-art image reconstruction techniques that infer missing data in a sparse acquisition space cannot recover the loss of spatial detail, especially at high temporal acceleration rates. The purpose of this paper is to introduce the concept of spatial subspace reconstructions (SPARS) and demonstrate its ability to reconstruct *high spatial resolution* dynamic images from as few as one acquired k-space spoke per time frame in a dynamic series. Briefly, a low-temporal-high-spatial resolution organization of the acquired raw data is used to estimate the basis vectors of the spatial subspace in which the high-temporal-high-spatial ground truth data resides. This subspace is then used to estimate entire images from single k-space spokes. In both simulated and human in-vivo data, the proposed SPARS reconstruction method outperformed standard GRASP and GRASP-Pro reconstruction, providing a shorter reconstruction time and yielding higher accuracy from both a spatial and temporal perspective.

## Introduction

Many biological and physiological phenomena are dynamic–flowing blood, beating heart, biochemical flux–and require equally dynamic techniques to study them. This requirement imposes stringent demands on whole-body imaging modalities such as MRI that are inherently slow but otherwise ideally suited to probing deep tissue with excellent contrast. The necessity for rapid imaging is well exemplified in DCE-MRI of microvascular function [1–5], where the temporal resolution must be one second or better to accurately capture the uptake dynamics of an intravenously injected contrast agent [6]. In practice, however, a far lower temporal resolution is used in exchange for the high spatial resolution and volume coverage needed in most DCE-MRI applications [6, 7]. This tradeoff comes at a significant cost: inaccurate quantitation of perfusion and other microvascular parameters, where such inaccuracy can compromise confidence in disease diagnosis or treatment monitoring.

Acceleration strategies have been proposed over the years to achieve both a high spatial resolution needed to detect small pathology *and* a high temporal resolution for accurate perfusion measurement. The earliest DCE-MRI-specific efforts implemented view sharing, which involved sampling the contrast-dense, low-frequency region of the acquisition domain (also known as k-space) more frequently than the high-frequency regions. These techniques, known as TWIST on Siemens scanners and TRICKS on General Electric, are widely available [8–10], but spatial resolution is suboptimal. Since then, more general methods, such as kt-BLAST [11] and the subsequent x-f choice [12], have been employed to accelerate perfusion imaging. Most recently, explorations into deep learning reconstruction of DCE-MRI data have emerged, but lack of large open-source databases for DCE-MRI presents a significant hurdle to advancing this technique beyond the proof-of-concept stage.

Perhaps the most established yet recent approach for reconstructing under-sampled DCE-MRI data is golden angle radial sparse parallel (GRASP) and a later version called GRASP-Pro [13–18]. GRASP outperformed previous acceleration methods, using radial sampling and compressed sensing (CS) with a total variation (TV) constraint for reconstruction. GRASP-Pro built on this foundation and further enforced the assumption that the true DCE-MRI data lay in a low-dimensional temporal subspace. Briefly, after performing a low spatial resolution GRASP reconstruction, the basis vectors for a temporal subspace were learnt through singular value decomposition or principal component analysis; all of k-space was then utilized for a high spatial resolution CS reconstruction, where the reconstruction was restricted to fall within the learned temporal subspace. As few as 13 radial spokes were sufficient to reconstruct 256×256 images. GRASP is not the only acceleration method that utilizes subspaces, however. In dynamic imaging, Otazo et al. and Ravishankar et al. constrained reconstructions to be comprised of individual sparse and low rank components [19, 20]. In MR spectroscopy, spatio-spectral correlation (SPICE) was developed to reconstruct high-spectral, high-spatial resolution spectroscopic imaging data from spectral basis vectors learned from a high-spectral, low-spatial resolution training scan [21, 22].

While subspaces can offer excellent reconstruction quality in accelerated scans, the type of subspace used–temporal or spatial–can heavily influence results. In GRASP, because reconstruction is constrained to fall in a temporal subspace, an underdetermined problem can emerge in the mid to high frequency regions of k-space, even when a perfect subspace is known (see S1 Appendix). This is solely a consequence of sparser sampling of higher frequency regions and less data for reconstruction, compared to lower frequency regions. If, however, we turn to a *spatial* subspace approach, we can overcome the problem of underdetermined optimization by encouraging the final reconstruction to fall near a learned spatial subspace. The use of spatial priors to accelerate a time series acquisition has been documented in the literature since 1988, beginning with the introduction of spectral localization by imaging (SLIM) [23], which used a high-resolution structural image as prior knowledge to enhance spectroscopic imaging. Spatial priors were also demonstrated for MR angiography using highly constrained back-projection (HYPR) [24]. In this work, we present the first demonstration of using spatial subspaces to reconstruct highly under-sampled DCE-MRI data. The superior performance of our reconstruction method is benchmarked against GRASP-Pro, the current reference for DCE-MRI acceleration.

## Theory

**Fig 1:**
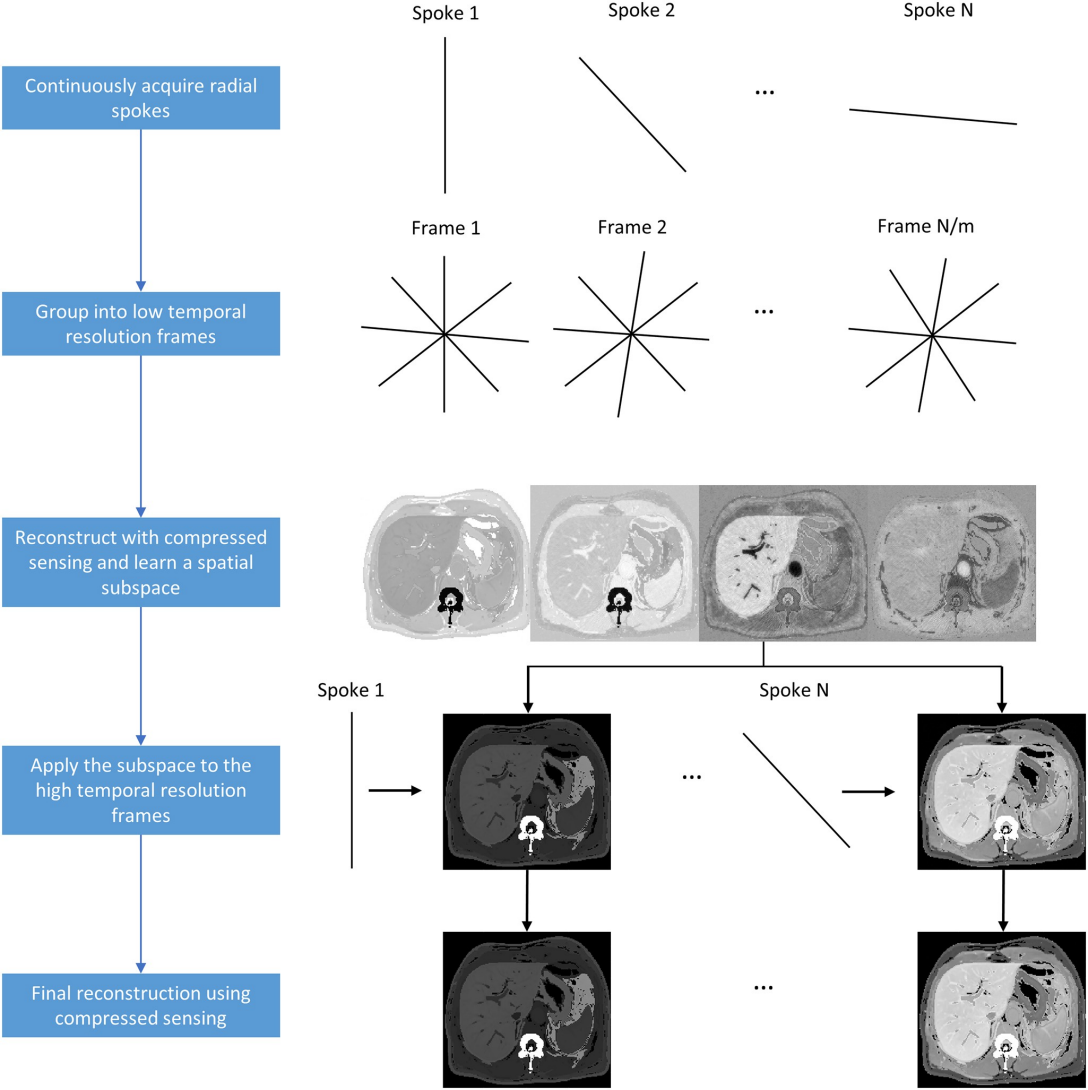
Flowchart of the reconstruction algorithm. Example is shown for reconstructing axial abdominal MRI images at successive timepoints.

**Fig 2:**
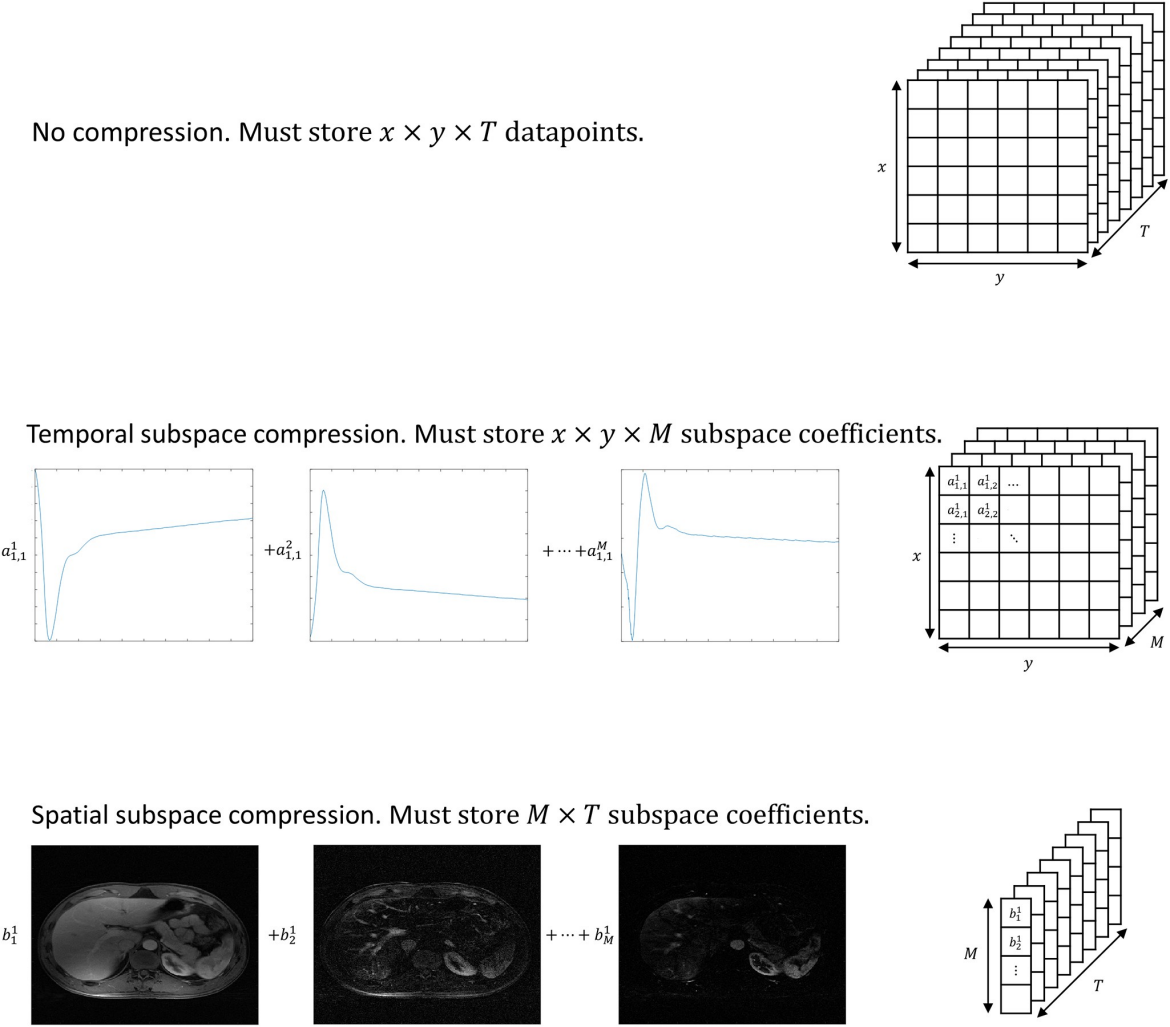
Comparison of compression via spatial versus temporal subspaces. It is assumed M subspace vectors are used to represent the dataset. Only the coefficients for the subspace vectors are considered. Top row: the format of the stored data for no compression. Middle row: each coefficient represents the weighting of a temporal subspace vector for a particular pixel. Each spatial location must have M coefficients, resulting in x*y*M total coefficients. Bottom row: each coefficient represents the weighting of a spatial subspace vector. Each temporal location must have M coefficients, resulting in M*T total coefficients. Note that we only count the number coefficients stored since these are what are learned in the final high-spatial, high-temporal resolution reconstructions of SPARS and GRASP-Pro.

Continuous radial acquisition creates an opportunity for adjusting temporal resolution at the time of image reconstruction, as fewer or more radial spokes can be utilized for reconstructing an image to achieve a higher or lower temporal resolution, respectively. Unfortunately, if a reconstruction needs to be performed with a high spatial resolution, as when the imaging target of interest is small, temporal resolution must be traded and what is attainable often does not permit accurate DCE-MRI measurements. However, we can step back from this notion that spatial and temporal resolutions are inexorably linked in DCE-MRI. Suppose we can determine the spatial subspace by identifying the basis vectors that capture the dominant spatial features of the dataset from a high spatial, low temporal resolution reconstruction. If each distinct anatomical feature can be accurately represented by the basis vectors of the spatial subspace, then it is possible to reconstruct a high spatial, high temporal resolution image from the same dataset. We dub this new approach Spatial-Subspace Reconstructions (SPARS) (Fig 1), where an image at each time point is reconstructed using a weighted combination of the most relevant subspace vectors describing the entire DCE-MRI dataset (S1 Fig shows reconstructions for different numbers of basis vectors). The difference in the number of coefficients one must estimate for the spatial subspace approach and for the conventional temporal subspace approach is illustrated in Fig 2. This figure does not account for the storage requirements of the subspace vectors, which are the basis vectors that span the subspace. It is crucial to understand that these subspace vectors must be estimated as part of the reconstruction process.

Consider a DCE-MRI dataset where images are of size N × N pixels and T radial spokes in total over the dynamic data set were acquired. The first step of SPARS is to perform a higher spatial resolution reconstruction by estimating a small number of temporal frames via solving the following equation:

$${\hat{\text{x}}}_{\text{L}}={\text{argmin}}_{{\text{x}}_{\text{L}}}\;∥{\text{Ex}}_{\text{L}}-{\text{y}}_{\text{L}}{∥}_{2}^{2}+\lambda ∥{\text{TV}}_{\text{time}}\left({\text{x}}_{\text{L}}\right){∥}_{1}+\gamma ∥{\text{x}}_{\text{L}}{∥}_{*}$$ (1)

where ${\hat{\text{x}}}_{\text{L}}\in {\mathbb{C}}^{{\text{N}}^{2}\times \frac{\text{T}}{\text{L}}}$ is the estimated reconstruction at L spokes per frame, with each estimated image vectorized as a column in the matrix (a Casorati matrix), and x_L_ is a candidate estimate for ${\hat{\text{x}}}_{\text{L}}$. E = ΦFB is an encoding operator that includes three operations: Φ is k-space under-sampling, F is a non-uniform fast Fourier transform (NUFFT), and B is an operator accounting for coil sensitivities. y_L_ are the vectorized and temporally sorted under-sampled measurements at L spokes per frame. TV_time_ applies temporal total variation, which involves calculating the gradient (or rate of change) of the image intensities over time in the temporal direction. The term ‘numerical gradient’ here refers to the computed differences in pixel intensities between consecutive time points. ‖∙‖_*_ is the nuclear norm (an L1 norm on the singular values of x). λ and γ are regularization parameters for the L1 temporal TV and nuclear norm, respectively. The purpose of ${\hat{\text{x}}}_{\text{L}}$ is to provide enough information to estimate the basis vectors of the spatial subspace in which a higher temporal resolution reconstruction is hypothesized to lie. We hypothesize that the distinct signal enhancement patterns of individual anatomical structures are captured in both high and low temporal resolution reconstructions. Therefore, both high and low temporal reconstructions are assumed to reside in the same spatial subspace. This hypothesis is later supported in the simulation results. An in-depth discussion of this hypothesis, its implications, and its limitations is found in the Discussion section.

Singular value decomposition is performed next on the output of Eq [1]. The b most significant basis vectors are retained and stored in ${\text{U}}_{\text{b}}\in {\mathbb{C}}^{{\text{N}}^{2}\times \text{b}}$. Then, the acquired data is re-organized to H spokes per frame with H < L (and, thus, the total number of frames increased), and a high-temporal resolution reconstruction is performed by solving the following equations:

$${\hat{\text{x}}}_{\text{H}}={\text{U}}_{\text{b}}{\hat{\text{A}}}_{\text{H}}$$ (2)

$${\hat{\text{A}}}_{\text{H}}={\text{argmin}}_{{\text{A}}_{\text{H}}}\;∥{\text{EU}}_{\text{b}}{\text{A}}_{\text{H}}-{\text{y}}_{\text{H}}{∥}_{2}^{2}+\lambda ∥{\text{TV}}_{\text{time}}\left({\text{U}}_{\text{b}}{\text{A}}_{\text{H}}\right){∥}_{\text{p}}$$

where ${\hat{\text{x}}}_{\text{H}}\in {\mathbb{C}}^{{\text{N}}^{2}\times \frac{\text{T}}{\text{H}}}$ is a high temporal resolution estimate of the dataset. ${\hat{\text{A}}}_{\text{H}}\in {\mathbb{C}}^{\text{b}\times \frac{\text{T}}{\text{H}}}$ is the matrix of coefficients corresponding to the optimal weights of each basis vector, and A_H_ is a candidate for ${\hat{\text{A}}}_{\text{H}}$. y_H_ are the vectorized and temporally sorted measured k-space spokes at H spokes per frame. TV_time_ performs temporal total variation as defined earlier, λ is the regularization parameter for the temporal regularization, and p determines the norm used. The S1 Appendix discusses why spatial subspaces are preferred over temporal subspaces.

## Materials and methods

### Simulated and in-vivo datasets

Two single-channel datasets were synthesized to resemble an axial MRI cross-section of an abdomen and brain. This was done to demonstrate that the algorithm was not specific to either of the two simulated datasets and was generalizable. To simulate the datasets, generic axial MRI images of a human abdomen (https://www.siemens-healthineers.com/en-us/magnetic-resonance-imaging/clinical-media/sola0016.html) and brain (https://radiopaedia.org/images/15543906) were used. These images were manually segmented based on knowledge of anatomy, and each organ was assigned its literature values for longitudinal relaxation time (*T*_1_) and pharmacokinetic (PK) parameters, namely, the transfer constant (*K*^*trans*^), extravascular-extracellular volume fraction (*v*_*e*_), and plasma volume fraction (*v*_*p*_). In partitioning anatomy into different tissue types, some pixels were not assigned to anatomy and were given arbitrary PK parameter values. The literature values assigned to each anatomy can be found in S1 Table. S2 Fig shows the labelled anatomy of the abdomen and brain used for simulation. S3 and S4 Figs show the exact assignment of pixels to anatomy. From these literature values, full resolution images of size 200×200 were generated over 1000 temporal frames, assuming the concentration-versus-time curve adhered to the Tofts model and assuming the following parameters for acquisition and contrast agent administration: longitudinal relaxivity *r*_1_ = 4.1 *L mmol*^−1^ *s*^−1^, repetition time (TR) = 6.2 ms, flip angle (FA) = 20°, contrast agent dose = 0.05 mmol/kg. The temporal resolution of the dataset was 0.18 seconds per radial spoke. We assumed an arterial input function (AIF) described by the population average from Parker et al. [25]. To evaluate each method in an ideal scenario, no noise was added to the simulated data. It was assumed that there was no change in signal intensity aside from that induced by a bolus injection.

In-vivo cross-sectional liver data from two human subjects provided in the public domain by Feng et al. and previously published in *Magnetic Resonance in Medicine* was used to validate SPARS [16, 26]. Both datasets were acquired on MAGNETOM 3 Tesla clinical scanners. The first dataset contains 600 spokes, 768 samples per readout, and 12 channels acquired with a FLASH sequence; the second dataset contains 1100 spokes, 512 samples per readout, and 20 channels acquired with a bSSFP sequence. Full details for the scans and calculation of the coil sensitivity maps can be found in Feng et al. 2014 [16] and Feng et al. 2018 [26]. The raw data and GRASP reconstruction algorithm code are available for download at https://cai2r.net/resources/xd-grasp-matlab-code/ and https://cai2r.net/resources/grasp-matlab-code/.

### Reconstruction of simulated data

To simulate realistic continuous radial acquisition, the first frame and last (i.e. 1000^th^) frame were simulated without undersampling (i.e. k-space is fully sampled for these frames). This assumption that the first and last frame can be sampled fully is reasonable, as the first frame represents pre-contrast injection (no contrast enhancement has yet occurred, so rapid acquisition is not required) and the last frame represents a relatively stable portion of contrast washout (where longer imaging to acquire the full k-space is permitted). For each of the remaining 998 frames, k-space was under-sampled using a NUFFT, retaining only one radial spoke per frame; the angle between the spokes of any two successive frames was set at the golden angle (~137.5°), in a manner similar to GRASP. To generate the low temporal resolution reconstruction, Eq [1] was then solved with *L* = 25, $\lambda =0.25\;{\text{max}}_{x\in {\text{E}}_{\text{H}}y}\;\left|x\right|$, and $\gamma =0.5\;{\text{max}}_{x\in {\text{E}}_{\text{H}}y}\;\left|x\right|$, which were empirically chosen based on reconstruction quality assessed using mean squared error (MSE). Here, the E_H_ operator is an inverse zero-filling NUFFT. These parameters were chosen from a pool of candidate combinations based on their ability to reconstruct data on a separate set of ground truth data. The term ${\text{max}}_{x\in {\text{E}}_{\text{H}}y}\;\left|x\right|$, which selects the maximum absolute value from the zero-filled, inverse Fourier transform of the observed data, ensures that these multipliers are chosen relative to the magnitude of the observed data. Eq [2] was then solved with λ = 0 to test the ability of the algorithm to reconstruct in the absence of noise and temporal regularization.

For comparison, GRASP-Pro was also implemented. However, rather than estimating a temporal subspace as per the first step of the GRASP-Pro algorithm, we provided the reconstruction with ground-truth, not estimated, temporal subspace vectors (i.e. temporal subspace vectors acquired from a full resolution, fully sampled ground truth dataset). This was done intentionally to give GRASP-Pro the greatest advantage possible, namely, having at its disposal a perfectly estimated temporal subspace. Three implementations of GRASP-Pro were evaluated: 5, 7, and 16 temporal basis vectors for reconstruction. These numbers were chosen to span a wide range of possible dataset dimensionalities, with 16 exceeding the required number of subspace vectors for data representation and 5 being the minimum number to represent the datasets without incurring substantial data loss.

### Reconstruction of in-vivo data

Eq [1] was solved with *L* = 21, $\lambda =0.25\;{\text{max}}_{x\in {\text{E}}_{\text{H}}y}\;\left|x\right|$, and $\gamma =0.5\;{\text{max}}_{x\in {\text{E}}_{\text{H}}y}\;\left|x\right|$ for the first dataset, and with *L* = 34, $\lambda =0.25\;{\text{max}}_{x\in {\text{E}}_{\text{H}}y}\;\left|x\right|$, and *$\gamma =0.5\;{\text{max}}_{x\in {\text{E}}_{\text{H}}y}\;\left|x\right|$* for the second dataset. Then, Eq [2] was solved with *H* = 1, $\lambda =50\;{\text{max}}_{x\in {\text{E}}_{\text{H}}y}\;\left|x\right|$, and p = 2. For each dataset, *L* was chosen to be the lowest number that provides a good reconstruction. *L* is higher for the second dataset due to the increased spatial resolution of the dataset.

For comparison, a GRASP-Pro reconstruction was also performed on both datasets at 1 spoke per frame. The initial low-spatial resolution reconstruction was performed to produce images of 96×96 pixels. On this low spatial resolution reconstruction, singular value decomposition was performed to determine the five most important temporal subspace vectors. Full spatial resolution reconstructions were then performed and constrained to fall within the learned subspace.

## Results

All simulations used the same conjugate gradient method as in the original GRASP paper. This code was made publicly available by Feng et al [16]. All computation was performed with MATLAB 2022b on an AMD Ryzen 5700x processor.

### Simulated data

**Fig 3:**
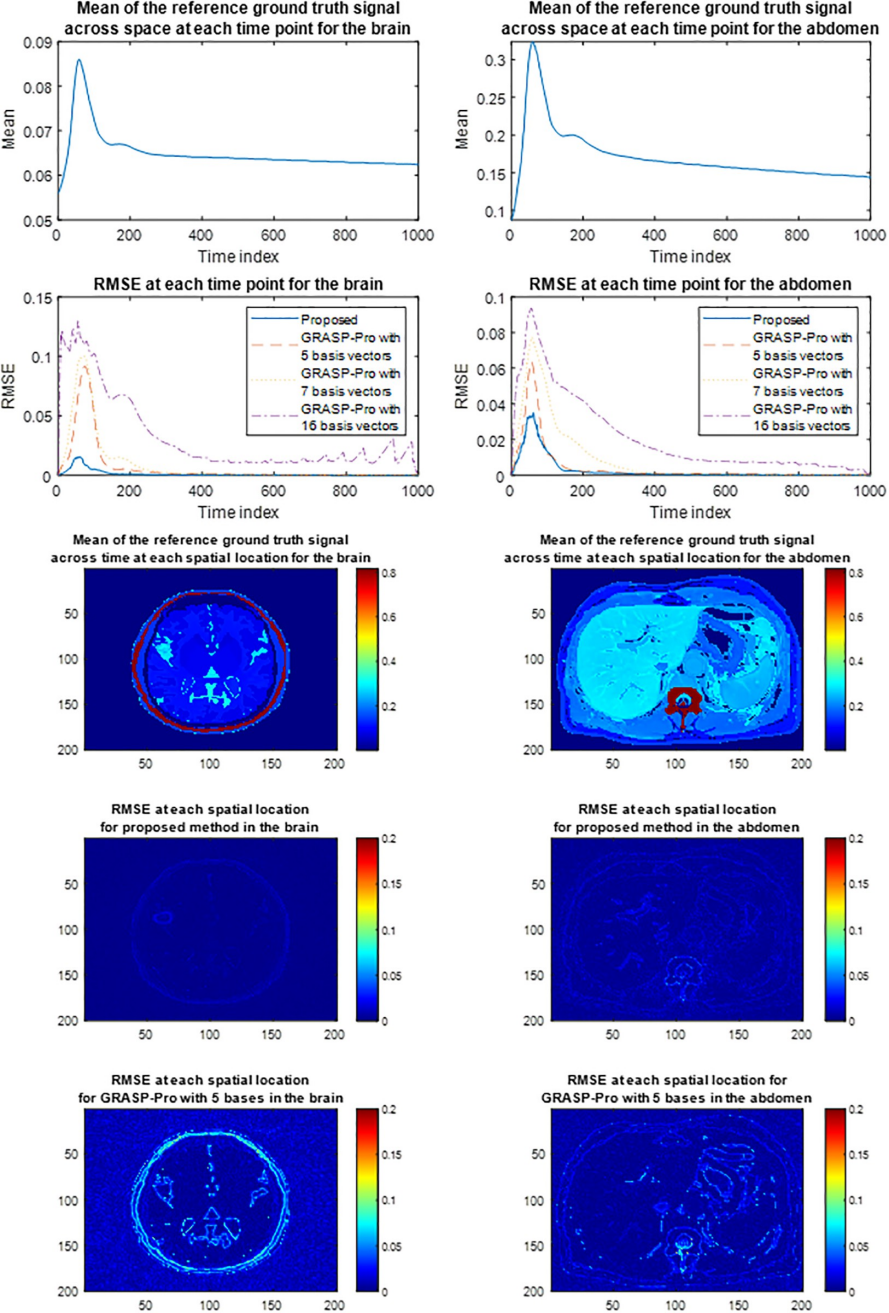
Comparison of reconstruction RMSE’s from proposed spatial subspace approach (SPARS) versus GRASP-Pro. RMSE at each point in time over all space (row 2) and at each point in space over all of time (rows 4 and 5). The simulated brain dataset is on the left, and the simulated abdomen dataset is on the right. For reference, the mean of the ground truth signals across all of space at each point of time is shown in row 1, and across all of time for each point in space in row 3.

**Fig 4:**
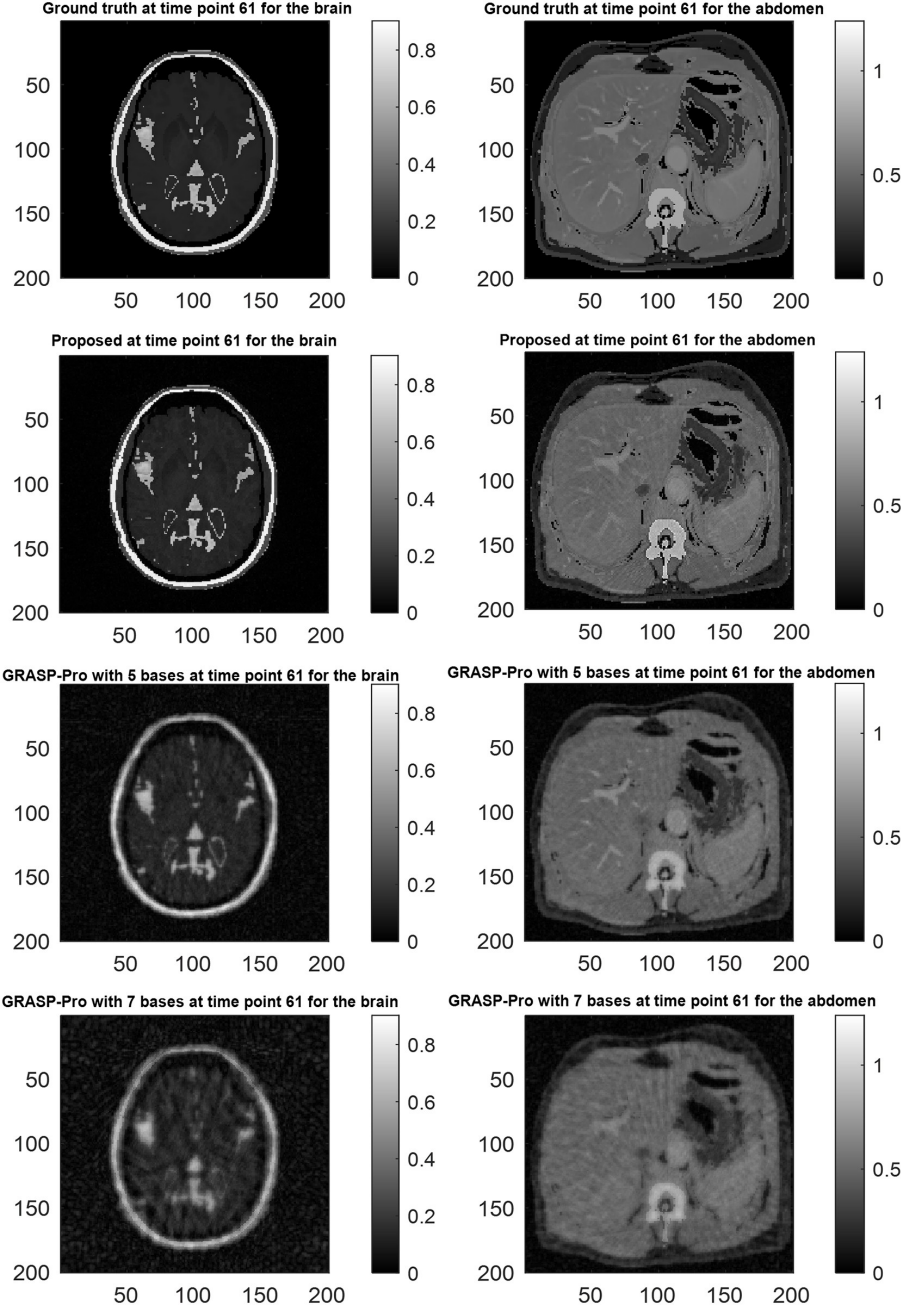
Comparison of reconstructed images from proposed spatial subspace approach (SPARS) versus GRASP-Pro. Reconstructions are shown for the time point corresponding to the highest error in SPARS (row 2) in the brain (left) and abdomen (right).

**Fig 5:**
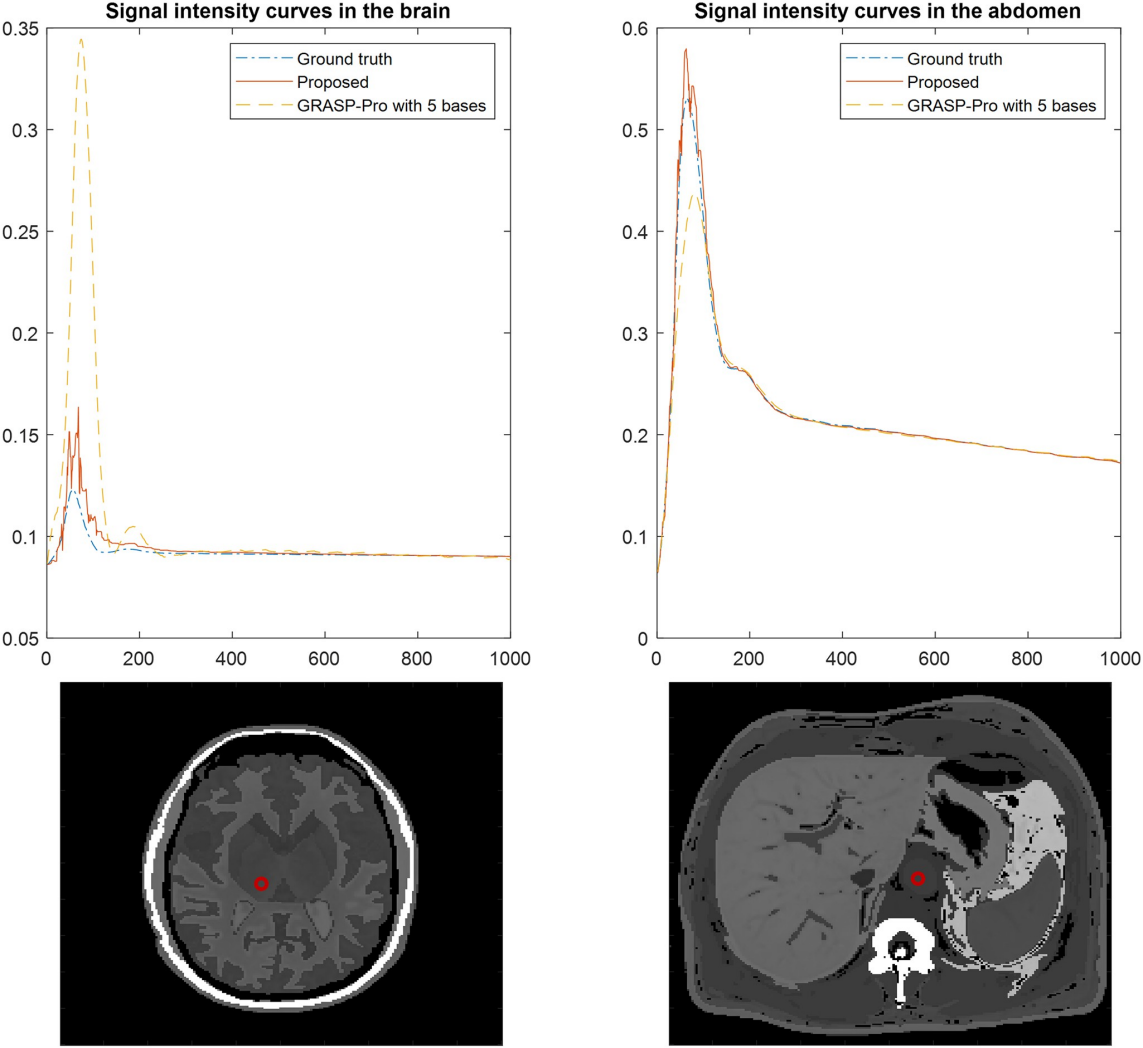
Comparison of estimated DCE-MRI signal-time curves for the proposed spatial subspace approach (SPARS) versus GRASP-Pro with perfect temporal subspace information. The ground truth DCE-MRI is shown for comparison in the brain at a single pixel in the thalamus (left) and in the abdomen at a single pixel in the aorta (right).

**Fig 6:**
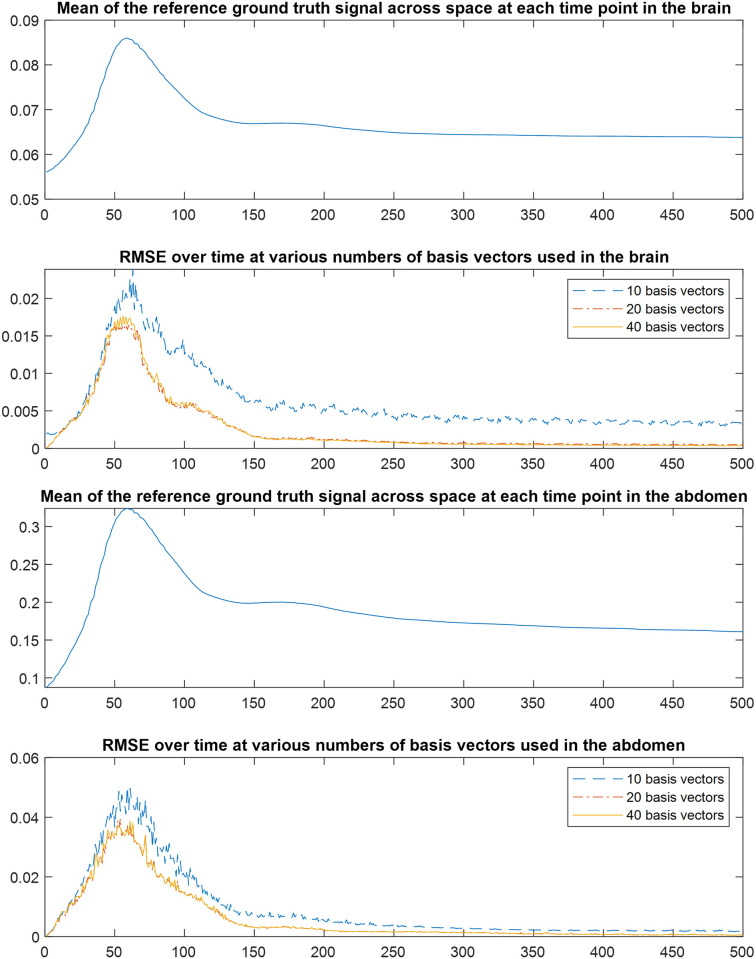
Reconstruction RMSE for SPARS as a function of the number of basis vectors. The RMSE over all space for the first 500 time points in the brain (row 2) and abdomen (row 4) of simulated reconstructions using 10, 20, and 40 estimated basis vectors. Only the first 500 time points are used, because the error does not increase in the last 500 time points. For reference, the mean of the ground truth signal across all of space at each point in time are shown in rows 1 and 3.

For the simulated datasets, both SPARS and GRASP-Pro were allowed to run until convergence when compressed sensing was used. This afforded each algorithm the highest chance of success independent of run time. In both cases, the total reconstruction time was approximately 10 hours. Note that this does not include the time to estimate the temporal subspace vectors for GRASP-Pro, since these were provided to the algorithm as described earlier. Reconstruction quality from an RMSE perspective is shown in Fig 3. The RMSE, whether viewed over time or over space, shows that SPARS offered more accurate reconstructions relative to GRASP-Pro. The largest errors were incurred along spatial edges in GRASP-Pro, whereas SPARS suppressed noise well in low and high spatial frequency domains. The improvement can also be appreciated by noting the better definition of high spatial frequency content (i.e. edges and small structures) from SPARS compared to GRASP-Pro (Fig 4). Finally, Fig 5 illustrates two examples of ground truth DCE-MRI signal intensity-time curves compared to estimates by our method and GRASP-Pro. One may notice that from an approximate time point of 150 onwards, the RMSE drops significantly. This is likely because, beyond this time point, the rate of signal intensity change begins to slow, typically as the contrast agent distribution stabilizes. This slower rate of change makes it easier for the reconstruction algorithm to capture the dynamics accurately. The effect of the number of basis vectors was also investigated. Fig 6 shows RMSE-over-time of reconstructions from Eq [2] using b values of 10, 20, and 40. A full analysis of total RMSE versus number of basis vectors is shown in S2 Table.

### In-vivo data

The compressed sensing routine ran for 27 iterations of conjugate gradient descent when solving Eq [1], and for 6 iterations when solving Eq [2]. The time taken to estimate the basis vectors and perform the final reconstructions is summarized in Tables 1 and 2.

**Table 1:** Comparing times for estimating subspace basis vectors and final reconstruction for the first in-vivo dataset.

Method	Subspace estimation time (spatial for SPARS, temporal for GRASP-Pro)	Final reconstruction time
GRASP-Pro	2870 s	21058 s
SPARS	749 s	466 s

**Table 2:** Comparing times for estimating subspace basis vectors and final reconstruction for the second in-vivo dataset.

Method	Subspace estimation time (spatial for SPARS, temporal for GRASP-Pro)	Final reconstruction time
GRASP-Pro	7421 s	25031 s
SPARS	929 s	667 s

**Fig 7:**
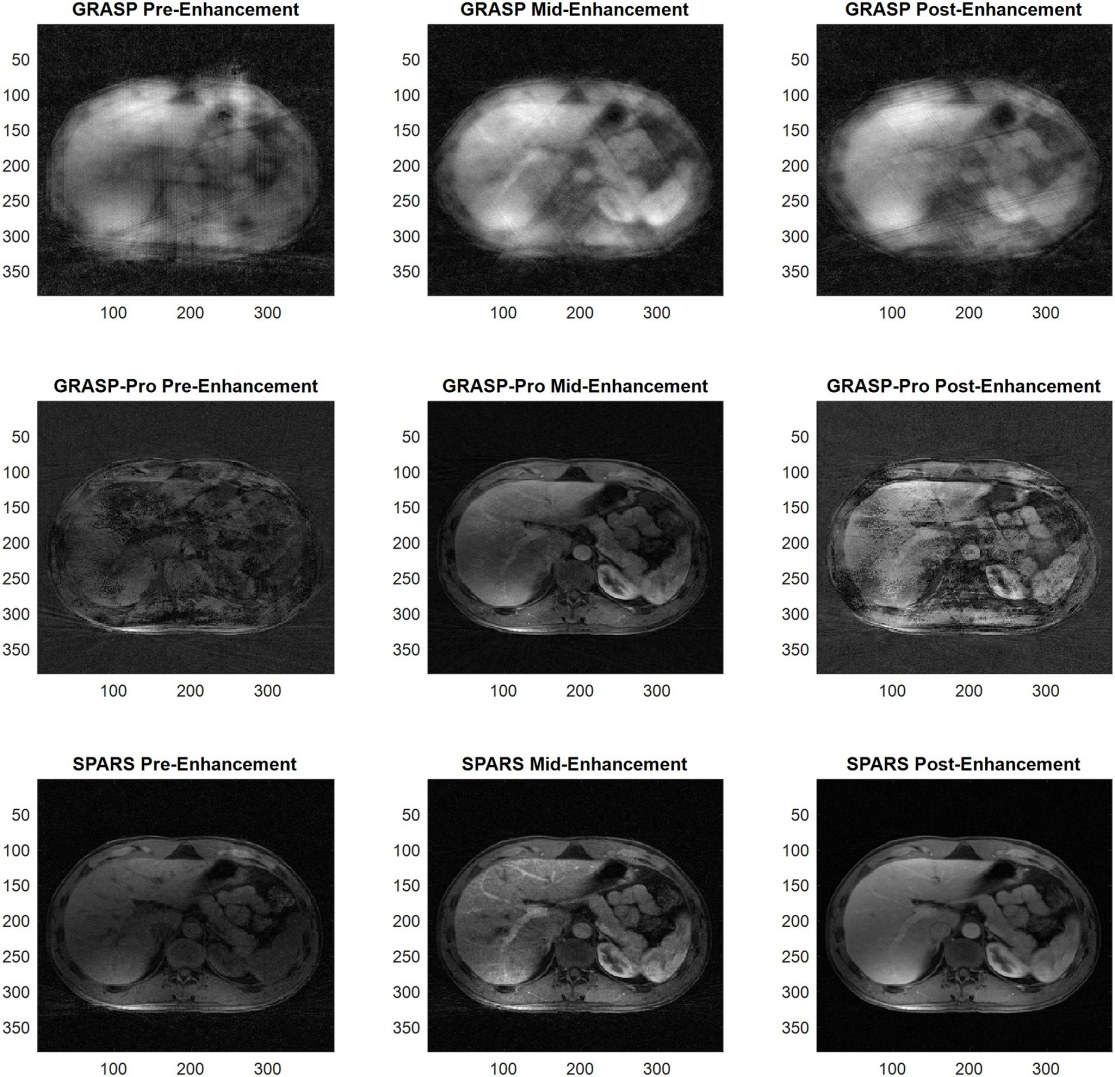
Reconstructed images of in-vivo human liver DCE-MRI for dataset 1. Comparison of GRASP, GRASP-Pro, and SPARS. Select timepoints are shown before, during, and after signal enhancement.

**Fig 8:**
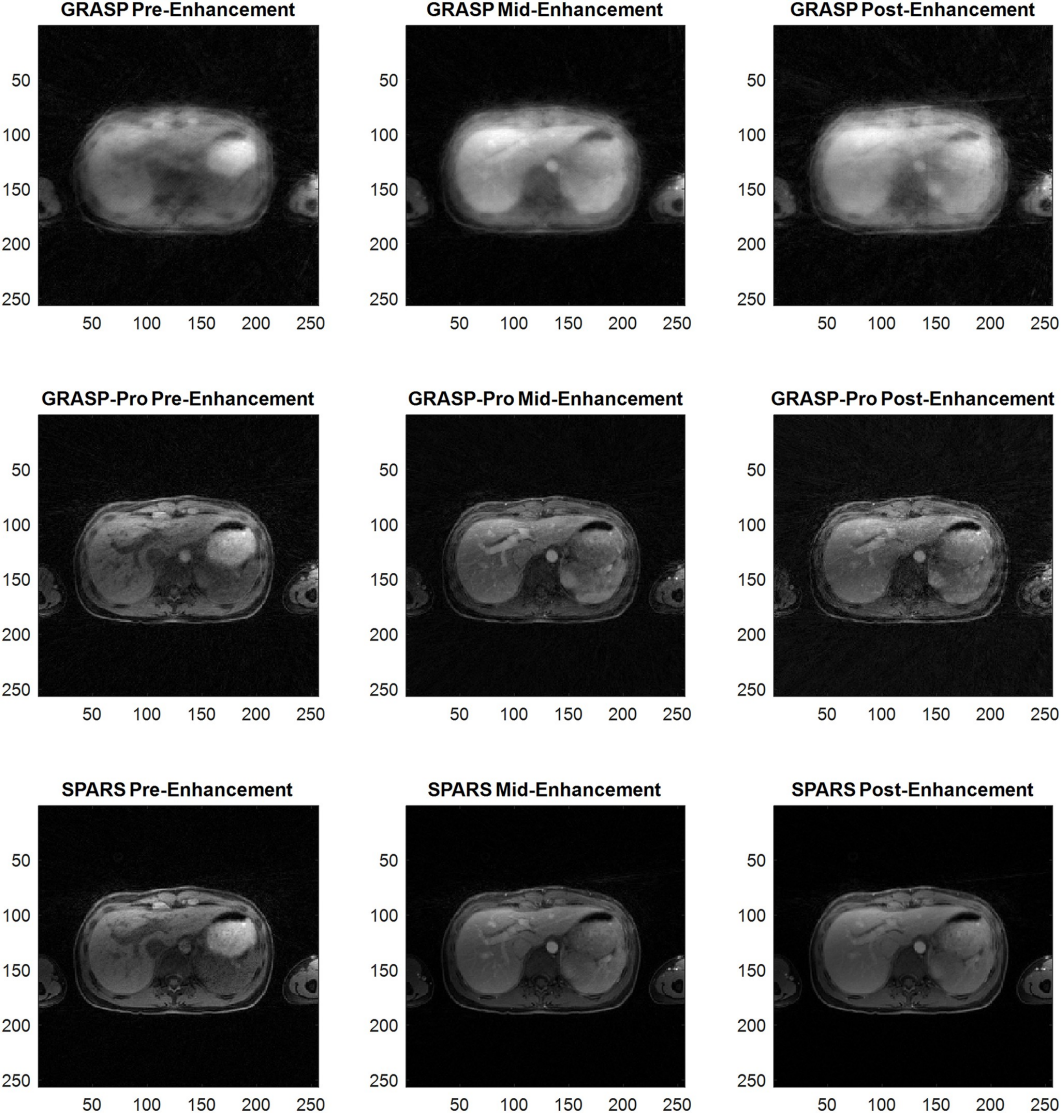
Reconstructed images of in-vivo human liver DCE-MRI for dataset 2. Comparison of GRASP, GRASP-Pro, and SPARS. Select timepoints are shown before, during, and after signal enhancement.

**Fig 9:**
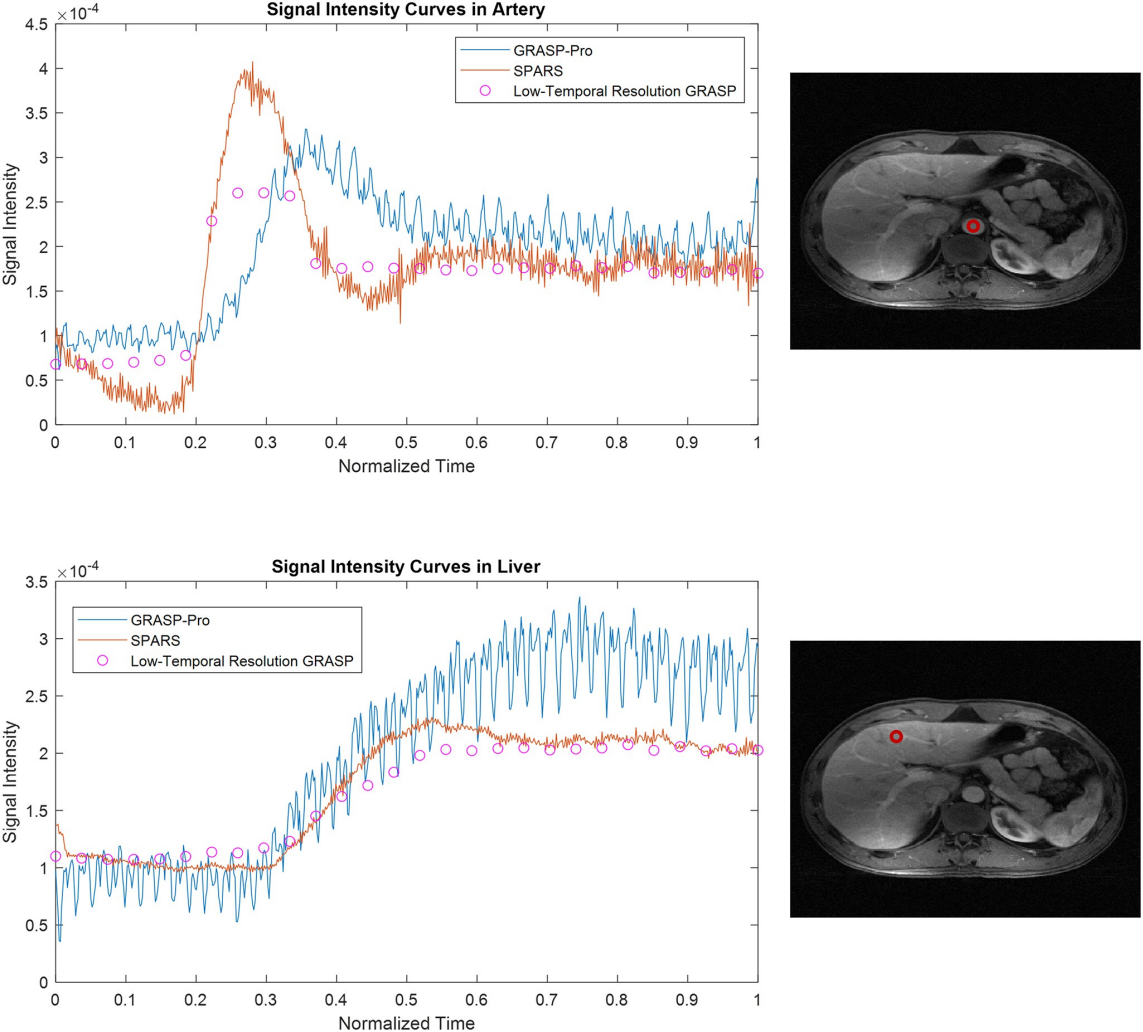
Temporal liver and aorta dynamics from in-vivo DCE-MRI reconstructions for dataset 1. Comparison of signal-time curves for GRASP-Pro, SPARS, and a low-temporal resolution GRASP reconstruction.

**Fig 10:**
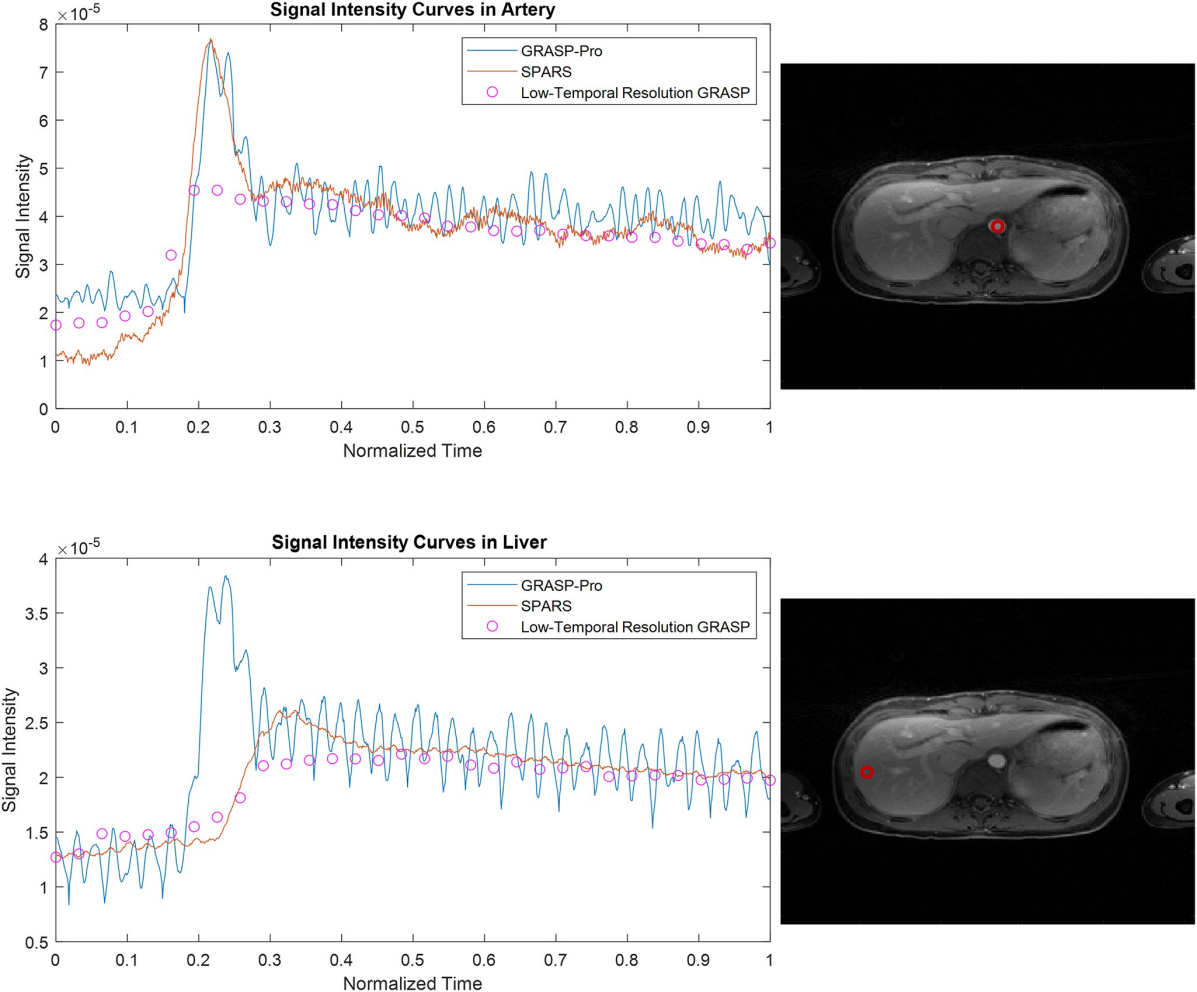
Temporal liver and aorta dynamics from in-vivo DCE-MRI reconstructions for dataset 2. Comparison of signal-time curves for GRASP-Pro, SPARS, and a low-temporal resolution GRASP reconstruction.

Figs 7 and 8 compare images reconstructed by SPARS versus GRASP-Pro, as well as images reconstructed with standard GRASP at 2 spokes per frame. Two spokes per frame was chosen for GRASP, as reconstruction at 1 spoke per frame did not complete in a reasonable time (>100,000 seconds). Figs 9 and 10 show the signal intensity over time at a location in the liver and in the artery for GRASP-Pro, SPARS, and a low-temporal resolution reconstruction of the same data. Although the low-temporal resolution signal intensity-over-time curve does not serve as ground truth, it does provide a time-averaged behavior of the signal dynamics. As seen in Fig 9, not only does GRASP-Pro suffer from worse signal corruption, but it also deviates farther from the low-temporal resolution dataset than does SPARS in both the artery and liver. This indicates that SPARS may be more representative of the true dynamic change in signal intensity over time. In the artery of the second dataset (Fig 10), GRASP-Pro and SPARS track the low-temporal resolution similarly, but GRASP-Pro suffers from worse noise corruption. In the liver of the second dataset, GRASP-Pro both deviates farther from the low-temporal resolution reconstruction and suffers from worse noise corruption.

S5 Fig shows the first 5 spatial basis vectors used by SPARS and the first 5 temporal basis vectors used by GRASP-Pro for each of the two in-vivo datasets. In the case of SPARS, each of the basis vectors contains distinct spatial information about the dataset. In the case of GRASP-Pro, the temporal basis vectors are corrupted by noise. This suggests that the temporal basis learned in the GRASP-Pro reconstruction may lack the comprehensive information required to accurately represent the high-temporal resolution signal enhancements present in the dataset.

## Discussion

We have described a novel reconstruction algorithm based on spatial subspaces to enable high-spatial and high-temporal resolution reconstruction of dynamic datasets, using as few as one acquired radial spoke in k-space to reconstruct a single image in a dynamic series. A spatial subspace is learnt from a low temporal resolution reconstruction and subsequently applied to a high temporal resolution reconstruction. As demonstrated, a learned spatial subspace provides more accurate reconstructions than a conventional temporal subspace. We believe the fundamental reason for the inferior performance of the latter lies in the intrinsic limitations of using temporal subspaces, described in greater detail in the S1 Appendix. Using fewer temporal subspace vectors results in a more over-determined (or well-determined) reconstruction in the higher frequencies (i.e. higher spatial resolution), but temporal dynamics are not as accurately modelled due to the reduced ability of the subspace to express them. In contrast, using fewer or more spatial subspace vectors will not affect the determinedness of the reconstruction problem so long as the number of vectors used for reconstruction is less than the number of k-space points sampled per frame. For example, if a particular frame has 100 sampled k-space points, then up to 100 spatial subspace vectors can be used for the reconstruction problem to remain well or over determined (more measured datapoints than variables to estimate, allowing for much more accurate fitting of the spatial subspace to the measured data). This property of spatial subspace reconstruction endows us with the flexibility to maintain both spatial and temporal resolution over a vast range in the number of spatial subspace vectors.

An additional advantage of a spatial subspace approach is the ability to supplement the subspace with knowledge acquired pre-contrast. For example, DCE-MRI examinations often involve the pre-contrast acquisition of a T_1_ map. The high spatial resolution images used to determine the T_1_ map can be included in the determination of the subspace at no additional cost and may provide extra anatomical information. This is not the case with temporal subspaces, because extra information on the temporal dynamics of signal enhancement cannot be acquired pre-contrast. Preservation of high spatial frequency details in the proposed spatial subspace reconstruction method is clear. In contrast, GRASP-Pro introduces severe blurring, and spatial resolution rapidly degrades with higher numbers of temporal basis vectors. Because the maximum number of temporal basis vectors for a well-determined reconstruction problem is generally low in the high frequency k-space areas (see S6 Fig), it is almost impossible not to exceed the optimal number in any practical implementation of GRASP-Pro. The higher the actual number of temporal basis vectors relative to the optimal, the more under-determined the optimization problem becomes. The faithful spatial representation afforded by SPARS also translated to faithful representation of temporal dynamics. Compared to GRASP-Pro, SPARS was able to follow more accurately ground-truth data, especially during the first-pass peak.

While the spatial subspace approach proposed in SPARS provides significant improvements in reconstruction quality, it is important to acknowledge that the fit of the spatial basis set may vary across different time points. This variability arises because the spatial subspace is learned from a low temporal resolution reconstruction, which may not fully capture rapid or transient signal changes occurring at specific time points. As a result, the accuracy of the reconstruction could be compromised during periods of rapid physiological change or motion. To mitigate this, future work could explore adaptive or hybrid methods that update the spatial basis set dynamically or integrate additional temporal information to ensure consistent accuracy across all time points.

Let us also delve deeper into the nature of signal corruption for in-vivo GRASP-Pro reconstruction. Our results reveal that GRASP-Pro could not properly estimate temporal subspace vectors from a highly undersampled, low-spatial resolution k-space dataset. This result is unsurprising–since the final reconstruction is determined solely as a linear combination of the temporal subspace vectors, which are estimated from undersampled measured data, any signal corruption in the measurement will propagate into the basis vectors and ultimately into the final reconstruction. One potential source of this signal corruption could simply be that the undersampling factor was too high for compressed sensing to produce an accurate reconstruction, even at the lower spatial resolution. This compounds the limitations of GRASP-Pro: first is the under-determinedness in the high frequency regions of k-space given any temporal subspace, and second is the ability to estimate the temporal subspace vectors themselves.

The effect of the number of basis vectors was also investigated. In both the brain and abdomen, using 20 basis vectors produced marginally better results than 40 basis vectors, and both were significantly better than a reconstruction using 10 basis vectors. The RMSE over time for a 10-basis-vector reconstruction was notably higher than that of the 20- or 40-basis-vector reconstructions. We also confirmed that all reconstructions with 20 or more basis vectors achieved similarly low RMSE. It is possible that vectors with the smallest singular values correspond to noise in the image and contribute marginally to the main object features.

Beyond demonstrating the superior reconstruction quality of spatial subspace over temporal subspace reconstruction, other considerations also determine reconstruction quality. One consideration is the number of basis vectors to use for SPARS; we found that basis vectors with the smallest singular values did not contribute to improved reconstruction quality, likely because they represent noise.

Tests on in-vivo human liver data acquired with free breathing confirmed the superiority of SPARS. Compared to GRASP-Pro, SPARS more faithfully captured high-spatial resolution details and true signal intensity temporal dynamics. For these reconstructions, temporal resolution can be on the order of 0.1 seconds per frame. While this may be higher than required for DCE-MRI, the purpose here is to demonstrate that SPARS can reconstruct more accurately than GRASP-Pro can, and that it can do so with fewer spokes, thereby potentially enabling imaging of larger volumes.

An additional advantage of SPARS is its short reconstruction time relative to GRASP-Pro. GRASP-Pro offers a per-slice reconstruction time of 20,000 to 30,000 seconds, whereas SPARS offers a per-slice reconstruction time of 1,000 to 1,500 seconds.

Finally, it is important to recognize that the success of SPARS depends on the accuracy with which a subspace learned from a low-temporal resolution reconstruction can represent a high-temporal resolution reconstruction. This may not always be the case. For example, small, transiently enhancing anatomical features may be missed in the low-temporal resolution reconstruction and these will be absent from the learned spatial subspace. One way to mitigate this issue is to increase the temporal resolution with which the spatial subspace is estimated. This can be done by realizing that Eq [1] can be replaced by any reconstruction method (e.g., GRASP-Pro). More generally, one can consider Eq [2] to be a supplement to any reconstruction technique. Another potential way to address this problem is to supplement the subspace with pre-contrast scans as mentioned earlier. In some cases, this may provide anatomical information that is not present in the dynamic scan.

## Conclusions

We proposed a novel spatial subspace (SPARS) estimation method to accurately reconstruct highly under-sampled DCE-MRI data. Our algorithm is shown to provide more accurate, higher spatial-resolution reconstructions than the current standard, GRASP-Pro, using only one radial acquisition spoke per time frame. A mathematical explanation was provided to elucidate the intrinsic difference between our spatial subspace estimation and conventional temporal subspace estimation: spatial subspaces are much more likely to result in an overdetermined optimization problem, whereas temporal subspace estimation often results in an underdetermined problem.

## Supporting information

S1 Fig Reconstruction using different numbers of basis vectors. Compression of a time point in a DCE-MRI series using different numbers of basis vectors.
(PDF)

S2 Fig Simulated abdomen (left) and brain (right) dataset. Native T1-weighted images pre-contrast are shown.
(PDF)

S3 Fig Components of simulated brain dataset. Pixels assigned to each type of brain anatomy are shown in white.
(PDF)

S4 Fig Components of simulated abdomen dataset. Pixels assigned to each type of abdominal anatomy are shown in white.
(PDF)

S5 Fig Comparison of subspace vectors used for in-vivo human liver DCE-MRI reconstructions. The first 5 learned spatial subspace vectors used in SPARS (rows 1 and 3) and the first 5 learned temporal subspace vectors used in GRASP-Pro (rows 2 and 4) for the two datasets.
(PDF)

S6 Fig Maximum number of temporal subspace vectors for well-determined reconstruction at each location in k-space. This figure shows the number of times k-space is sampled for 1000 spokes of a single coil, single nearest neighbor re-gridding of radially acquired k-space with each spoke separated by the golden angle. The color indicates the maximum number of temporal subspace vectors allowed for well or over-determined reconstruction.
(PDF)

S7 Fig Percentage of k-t space underdetermined for different numbers of temporal subspace vectors used for reconstruction by GRASP-Pro. Note the rapidly increasing number of k-space points that are underdetermined for 4 subspace vectors and higher.
(PDF)

S1 Table Parameters assigned to each organ for DCE-MRI simulations with the Tofts model. (PDF)

S2 Table Number of basis vectors used for L2 norm reconstruction and corresponding RMSE. For reference, the mean value across space and time is 0.065 for the brain dataset and 0.171 for the abdomen dataset.
(PDF)

S1 Appendix (DOCX)
