# Supplementary material for: Accelerated dynamic magnetic resonance imaging from Spatial-Subspace Reconstructions (SPARS)
Source: PLoS One. 2025 Jan 31;20(1):e0317271. doi: 10.1371/journal.pone.0317271 (PMC11785264; doi:10.1371/journal.pone.0317271)
Supplement: S1 Fig — Compression of a time point in a DCE-MRI series using different numbers of basis vectors. (PDF) [file pone.0317271.s001.pdf]

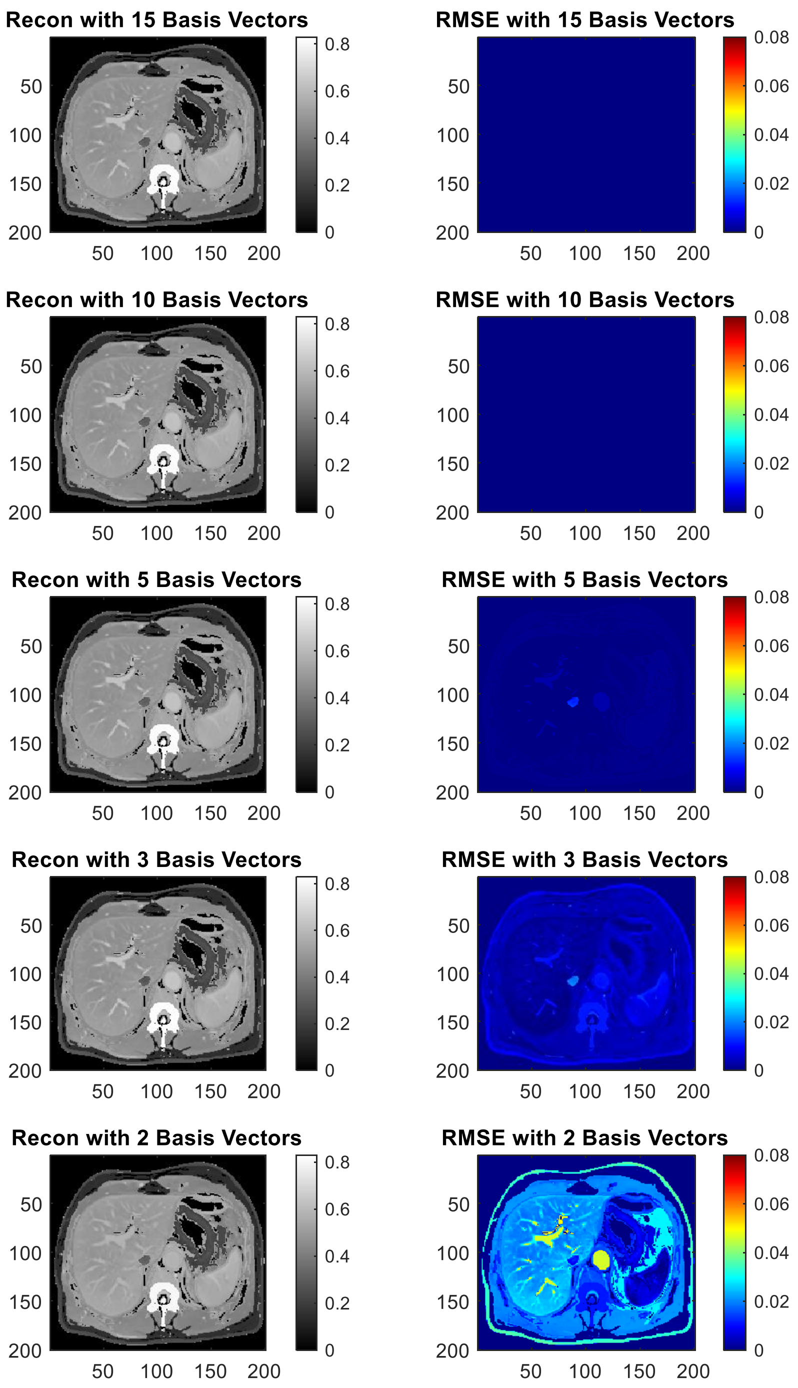

S1 Fig. Reconstruction using different numbers of basis vectors. Compression of a time point in a DCE-MRI series using different numbers of basis vectors.
