# Supplementary material for: Accelerated dynamic magnetic resonance imaging from Spatial-Subspace Reconstructions (SPARS)
Source: PLoS One. 2025 Jan 31;20(1):e0317271. doi: 10.1371/journal.pone.0317271 (PMC11785264; doi:10.1371/journal.pone.0317271)
Supplement: S2 Fig — Native T1-weighted images pre-contrast are shown. (PDF) [file pone.0317271.s002.pdf]

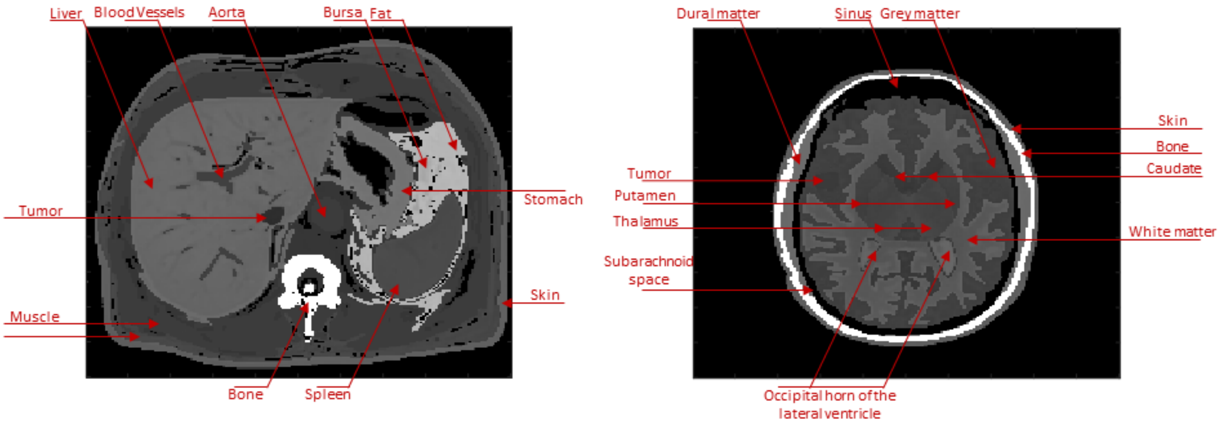

S2 Fig. **Simulated abdomen (left) and brain (right) dataset**. Native T1-weighted images pre-contrast are shown.
