# Supplementary material for: Accelerated dynamic magnetic resonance imaging from Spatial-Subspace Reconstructions (SPARS)
Source: PLoS One. 2025 Jan 31;20(1):e0317271. doi: 10.1371/journal.pone.0317271 (PMC11785264; doi:10.1371/journal.pone.0317271)
Supplement: S3 Fig — Pixels assigned to each type of brain anatomy are shown in white. (PDF) [file pone.0317271.s003.pdf]

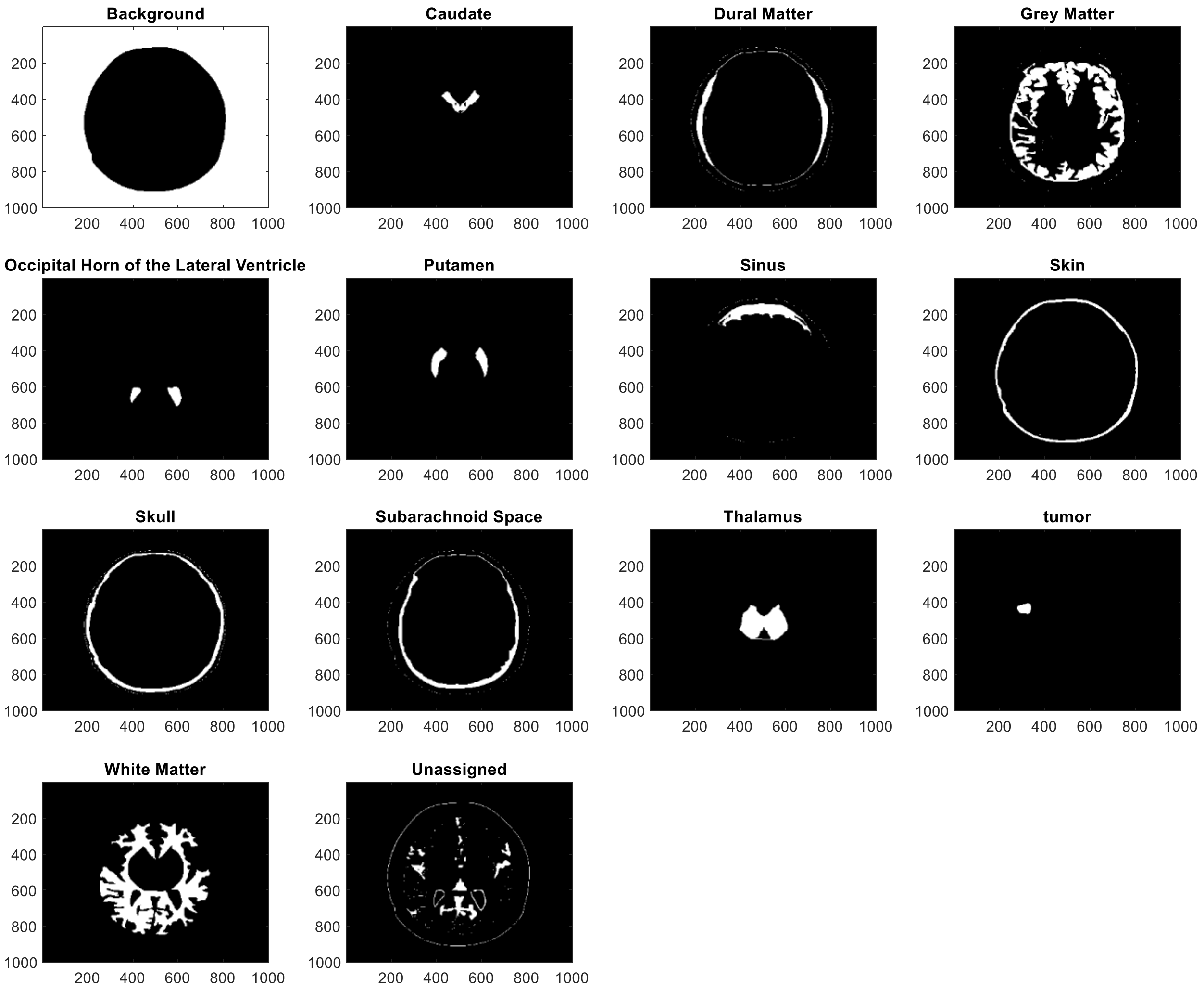

S3 Fig. Components of simulated brain dataset. Pixels assigned to each type of brain anatomy are shown in white.
