# Supplementary material for: Accelerated dynamic magnetic resonance imaging from Spatial-Subspace Reconstructions (SPARS)
Source: PLoS One. 2025 Jan 31;20(1):e0317271. doi: 10.1371/journal.pone.0317271 (PMC11785264; doi:10.1371/journal.pone.0317271)
Supplement: S5 Fig — The first 5 learned spatial subspace vectors used in SPARS (rows 1 and 3) and the first 5 learned temporal subspace vectors used in GRASP-Pro (rows 2 and 4) for the two datasets. (PDF) [file pone.0317271.s005.pdf]

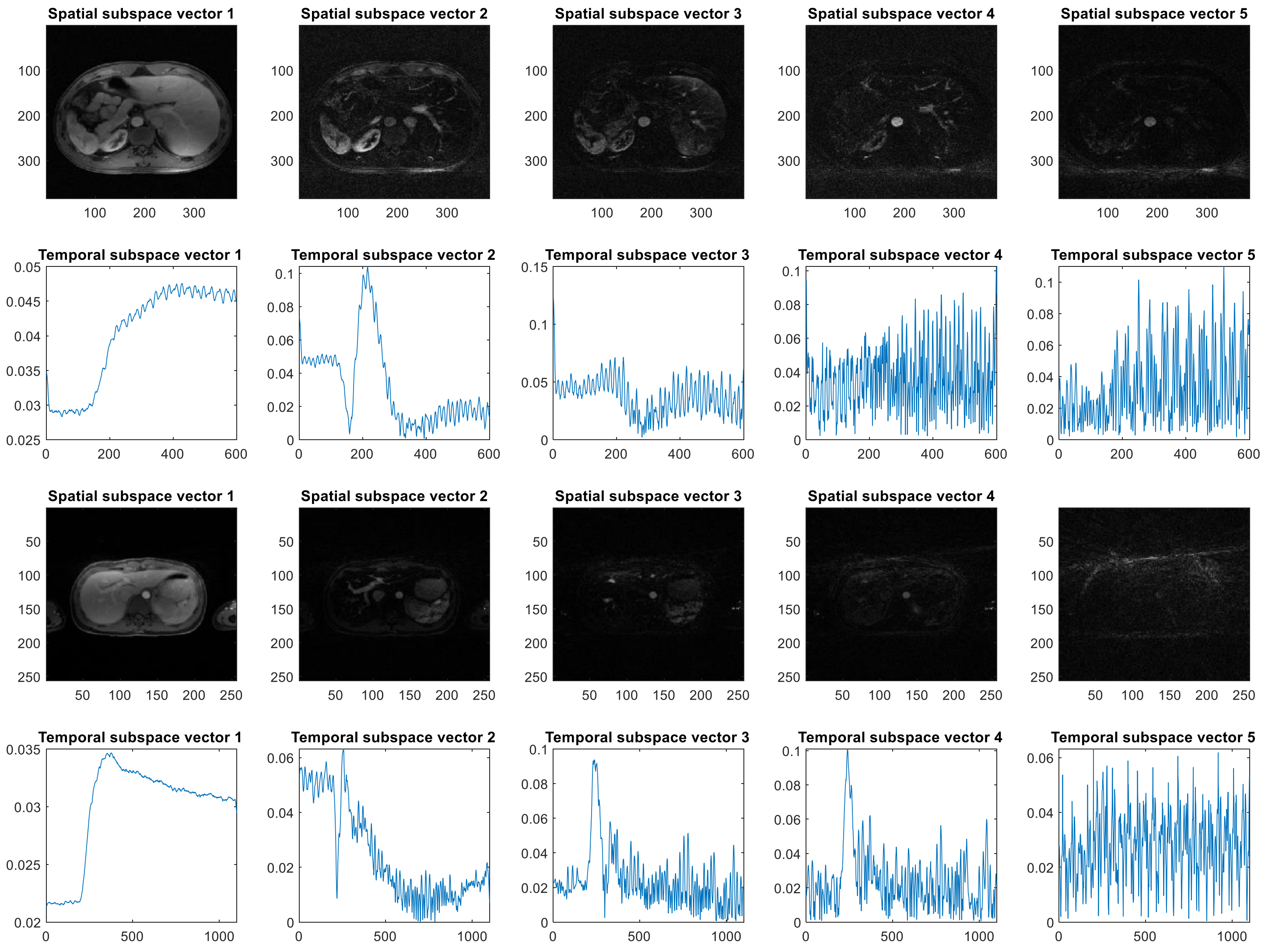

S5 Fig. Comparison of subspace vectors used for in-vivo human liver DCE-MRI reconstructions. The first 5 learned spatial subspace vectors used in SPARS (rows 1 and 3) and the first 5 learned temporal subspace vectors used in GRASP-Pro (rows 2 and 4) for the two datasets.
