# Supplementary material for: Accelerated dynamic magnetic resonance imaging from Spatial-Subspace Reconstructions (SPARS)
Source: PLoS One. 2025 Jan 31;20(1):e0317271. doi: 10.1371/journal.pone.0317271 (PMC11785264; doi:10.1371/journal.pone.0317271)
Supplement: S6 Fig — This figure shows the number of times k-space is sampled for 1000 spokes of a single coil, single nearest neighbor re-gridding of radially acquired k-space with each spoke separated by the golden angle. The color indicates the maximum number of temporal subspace vectors allowed for well or over-determined reconstruction. (PDF) [file pone.0317271.s006.pdf]

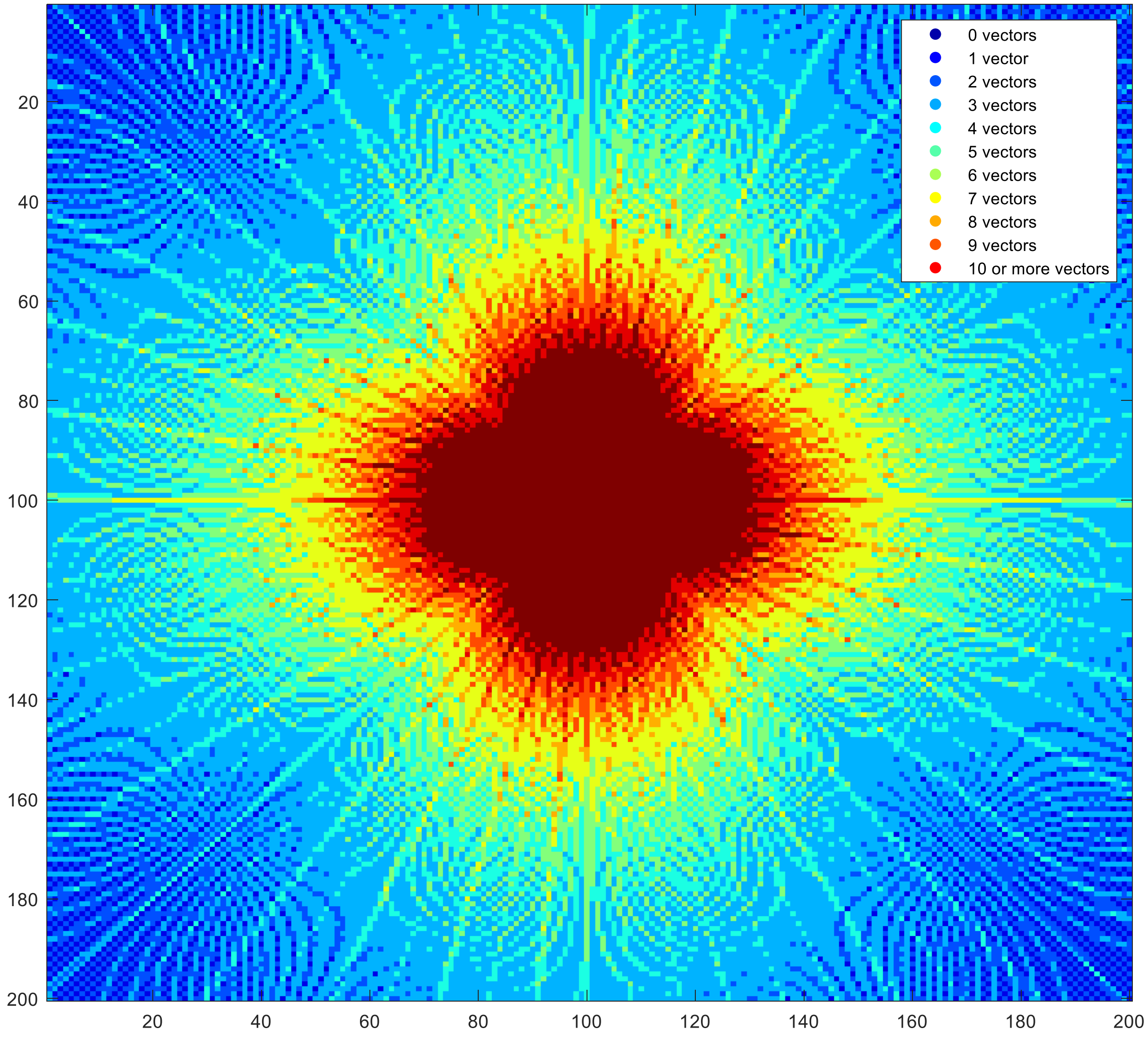

S6 Fig. Maximum number of temporal subspace vectors for well-determined reconstruction at each location in k-space. This figure shows the number of times k-space is sampled for 1000 spokes of a single coil, single nearest neighbor re-gridding of radially acquired k-space with each spoke separated by the golden angle. The color indicates the maximum number of temporal subspace vectors allowed for well or over-determined reconstruction.
