# Supplementary material for: Accelerated dynamic magnetic resonance imaging from Spatial-Subspace Reconstructions (SPARS)
Source: PLoS One. 2025 Jan 31;20(1):e0317271. doi: 10.1371/journal.pone.0317271 (PMC11785264; doi:10.1371/journal.pone.0317271)
Supplement: S7 Fig — Note the rapidly increasing number of k-space points that are underdetermined for 4 subspace vectors and higher. (PDF) [file pone.0317271.s007.pdf]

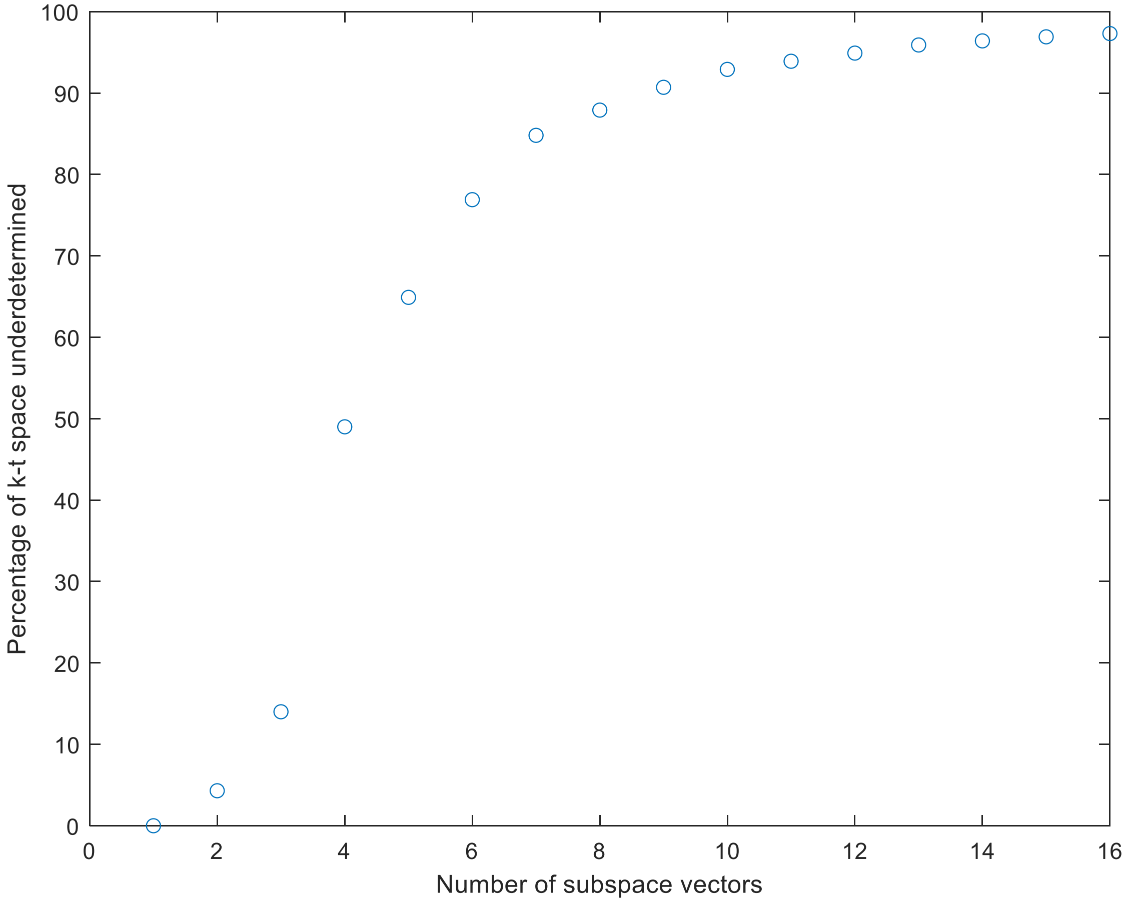

S7 Fig. Percentage of k-t space underdetermined for different numbers of temporal subspace vectors used for reconstruction by GRASP-Pro. Note the rapidly increasing number of k-space points that are underdetermined for 4 subspace vectors and higher.
