# Supplementary material for: Accelerated dynamic magnetic resonance imaging from Spatial-Subspace Reconstructions (SPARS)
Source: PLoS One. 2025 Jan 31;20(1):e0317271. doi: 10.1371/journal.pone.0317271 (PMC11785264; doi:10.1371/journal.pone.0317271)
Supplement: S1 Table — (PDF) [file pone.0317271.s008.pdf]

S1 Table. Parameters assigned to each organ for DCE-MRI simulations with the Tofts model

**Organ**	K^{trans} **range (min**$^{-1}$**)**	v_e **range**	v_p **range**	T_1 **range (ms)**
Skin	0.001 – 0.002	0 – 0.2	0.07 – 0.12	800 – 900
Spleen	3 – 4	0.2 – 0.35	0.2 – 0.29	1123 – 1323
Aorta	0	0	0.45 – 0.55	1750 – 1850
Liver	1 – 1.8	0.3 – 0.44	0.16 – 0.27	750 – 900
Liver Tumor	0.5 – 0.6	0.3 – 0.4	0.06 – 0.12	1800 – 2100
Liver Blood Vessels	0	0	0.45 – 0.55	1600 – 1700
Stomach	0.1 – 0.2	0.05 – 0.1	0.02 – 0.08	650 – 700
Bone	0.001 – 0.002	0.01 – 0.02	0.004 – 0.009	26
Fat	0.3 – 0.4	0.04 – 0.14	0.15 – 0.4	382 – 432
Bursa	0.03 – 0.07	0.04 – 0.06	0.01 – 0.03	380 – 450
Muscle	0.1 – 0.2, 0.4 – 0.6	0.05 – 0.2	0.02 – 0.04, 0.2 – 0.4	1100 – 1300, 1500 – 1650
Meningeal Dural Matter	0.004 – 0.005	0.05 – 0.15	0.02 – 0.04	700 – 800
Subarachnoid Space	0	0	0	300
Sinus	0	0	0	300 – 350
Brain Tumor	0.05 – 0.15	0.2 – 0.3	0.4 – 0.7	1800 – 2100
Grey Matter	0.0017 – 0.0019	0.024 – 0.026	0.017 – 0.03	1200 – 1400

**Organ**	K^{trans} **range (min^{-1})**	v_e **range**	v_p **range**	T_1 **range (ms)**
White Matter	0.0017 – 0.0024	0.021 – 0.027	0.01 – 0.02	800 – 1000
Occipital Horn of the Lateral Ventricle	0.002 – 0.005	0.02 – 0.17	0.01 – 0.02	750 – 950
Thalamus	0.0005 – 0.0015	0.17 – 0.25	0.015 – 0.037	1100 – 1300
Putamen	0.0003 – 0.0015	0.15 – 0.3	0.01 – 0.03	1200 – 1400
Caudate	0.003 – 0.0015	0.17 – 0.27	0.014 – 0.04	1400 – 1600
