# Supplementary material for: Accelerated dynamic magnetic resonance imaging from Spatial-Subspace Reconstructions (SPARS)
Source: PLoS One. 2025 Jan 31;20(1):e0317271. doi: 10.1371/journal.pone.0317271 (PMC11785264; doi:10.1371/journal.pone.0317271)
Supplement: S2 Table — For reference, the mean value across space and time is 0.065 for the brain dataset and 0.171 for the abdomen dataset. (PDF) [file pone.0317271.s009.pdf]

S2 Table. Number of basis vectors used for L2 norm reconstruction and corresponding RMSE. For reference, the mean value across space and time is 0.065 for the brain dataset and 0.171 for the abdomen dataset.

# Basis Vectors	Abdomen RMSE (10^{-2})	Brain RMSE (10^{-2})
5	1.23	0.773
10	0.328	0.572
15	0.286	0.324
20	0.0767	0.327
25	0.244	0.322
30	0.244	0.329
35	0.243	0.333
40	0.245	0.337
