# Supplementary material for: Accelerated dynamic magnetic resonance imaging from Spatial-Subspace Reconstructions (SPARS)
Source: PLoS One. 2025 Jan 31;20(1):e0317271. doi: 10.1371/journal.pone.0317271 (PMC11785264; doi:10.1371/journal.pone.0317271)
Supplement: S1 Appendix — (DOCX) [file pone.0317271.s010.docx]

**Appendix**

**The over- and under-determinedness of temporal and spatial subspaces**

We demonstrate in the following that SPARS will more frequently result in an over-determined reconstruction problem compared to GRASP-Pro. The proof lies in the fact, as shown below, that a very large number of spatial subspace vectors can be accommodated while still guaranteeing reconstruction remains an overdetermined problem. In contrast, a very low number of temporal subspace vectors needs to be maintained to guarantee the problem does not become underdetermined. For these reasons, it is theoretically possible to achieve for a spatial subspace an under-sampling rate greatly exceeding what is possible from a temporal subspace (given the same number of basis vectors).

We begin with SPARS. In the case of reconstruction with a spatial subspace, each point in time can be considered independently (i.e. each frame can be reconstructed independent of other frames), and the conditions for an overdetermined reconstruction problem change. With $b$ spatial subspace vectors and $M$ measured k-space points in a high-temporal-resolution frame, the condition required for the reconstruction problem to be overdetermined is:

$b\leq M$ (A1)

Let us explore this problem more deeply. Given a matrix, $U_{i}$, whose columns represent spatial subspace vectors, and a matrix, $A_{i}$, whose entries represent the coefficients used to reconstruct the dataset from the spatial subspace vectors, k-space can be formed using the following equation, where $y$ is the fully sampled k-space and $F$ is a Fourier transform matrix:

$y=FU_{i}A_{i}$ (A2)

Equation [A2] shows that to arrive at k-space for an image domain dataset, one can keep the same coefficients $A_{i}$and simply perform a 2D Fourier transform on the basis vectors in $U_{i}$. The determinedness of the problem remains unchanged, and equation [A1] remains sufficient for an overdetermined reconstruction.

Let us now examine temporal subspaces and how they are used for reconstruction, as in GRASP-Pro. For an $N\times N\times T$ dataset, where $N$ is the image dimension and $T$ is the number of time points, the resulting problem has $N\times N\times b$ variables to learn, where $b$ is the number of temporal subspace vectors. If $M$ is the number of measured k-space points in a high-temporal-resolution frame, the number of measured points is $T\times M$. Therefore, the weakest condition required to guarantee a well determined reconstruction problem is: $b\leq\frac{T\times M}{N\times N}$ (A3)

It is important to notice that SPARS is less restrictive than GRASP-Pro in the number of subspace vectors allowed to guarantee a well/over-determined reconstruction. This is especially true in the scenario where the number of time points is fewer than the number of pixels per frame, which is almost always true.

We now revisit more explicitly the reconstruction problem to tighten the upper bound given by equation [A3]. Given a matrix, $U_{t}$, whose columns represent temporal subspace vectors, and a matrix, $A_{t}$, whose entries represent the coefficients used to reconstruct the dataset from the temporal subspace vectors, k-space can be formed with the following equation, where $y$ is the fully sampled k-space and $F$ is a Fourier transform matrix:

$y=F\left( U_{t}A_{t} \right)^{\mathbf{T}}$ (A4)

After some manipulation, this can be expressed as:

$y=\left( U_{t}A_{t}F^{T} \right)^{\mathbf{T}}$ (A5)

Equation [A5] shows that the same temporal subspace used to construct the image-domain dataset can be used to construct the k-space-domain dataset. The only difference is in the coefficients used for reconstruction. The implication here is that with a temporal subspace, each point in k-space over the whole dataset can be reconstructed independently from every other point in k-space. When reconstructing under-sampled radial k-space data using a temporal subspace, points in k-space over time that are closer to zero spatial frequency (i.e. DC) will have more datapoints for reconstruction than those further away from DC. In other words, the DC point in k-space over time will be fully sampled, thus giving rise to an overdetermined reconstruction problem, whereas points further away from DC will have fewer datapoints for reconstruction and eventually give rise to an underdetermined reconstruction problem. Consequently, reconstruction with a temporal subspace is likely to be underdetermined in much of k-space over time; it is for this reason that CS reconstruction is required. This may be the case even when equation [A3] holds true. As seen in the Results section, GRASP-Pro reconstructions have less high spatial frequency data. Furthermore, **S6 Fig** illustrates at each point in k-space the maximum number of temporal subspace vectors allowed for reconstruction to remain well-determined or over-determined for simulated data. This figure was generated by simulating the acquisition of 1000 radial spokes separated by the golden angle on a Cartesian grid, and then counting the number of times each position on the grid is sampled across all 1000 spokes. The number of times a position in k-space is sampled determines the maximum number of allowed temporal subspace vectors for a well- or over-determined reconstruction. If more than the maximum number of temporal subspace vectors is used, the reconstruction problem will be under-determined. Notice in S5 Fig that the maximum number of temporal basis vectors decreases towards the higher spatial frequencies. At the highest spatial frequencies, this maximum number is between 0 and 2, which suggests that reconstructing high spatial frequencies using temporal subspace vectors will almost always be underdetermined. **S7 Fig** shows the percentage of k-space that is underdetermined for different numbers of temporal subspace vectors as obtained by S6 Fig.
